# Esophageal 3D organoids of *MPV17^-/-^* mouse model of mitochondrial DNA depletion show epithelial cell plasticity and telomere attrition

**DOI:** 10.18632/oncotarget.27264

**Published:** 2019-10-22

**Authors:** Manti Guha, Satish Srinivasan, Maura M. Sheehan, Takashi Kijima, Gordon Ruthel, Kelly Whelan, Koji Tanaka, Andres Klein-Szanto, Prasanna M. Chandramouleeswaran, Hiroshi Nakagawa, Narayan G. Avadhani

**Affiliations:** ^1^Department of Biomedical Sciences, School of Veterinary Medicine, University of Pennsylvania, Philadelphia, PA, USA; ^2^Division of Gastroenterology, Department of Medicine, University of Pennsylvania Perelman School of Medicine, Philadelphia, PA, USA; ^3^Histopathology Facility, Fox Chase Cancer Center, Temple University, Philadelphia, PA, USA; ^4^Herbert Irving Comprehensive Cancer Center, Columbia University Medical Center, New York, NY, USA

**Keywords:** mitochondrial DNA, *MPV17*, ESCC, telomere, 3D organoid

## Abstract

Esophageal squamous cell carcinoma (ESCC) is an aggressive cancer with late-stage detection and poor prognosis. This emphasizes the need to identify new markers for early diagnosis and treatment. Altered mitochondrial genome (mtDNA) content in primary tumors correlates with poor patient prognosis. Here we used three-dimensional (3D) organoids of esophageal epithelial cells (EECs) from the *MPV17^-/-^* mouse model of mtDNA depletion to investigate the contribution of reduced mtDNA content in ESCC oncogenicity. To test if mtDNA defects are a contributing factor in ESCC, we used oncogenic stimuli such as ESCC carcinogen 4-nitroquinoline oxide (4-NQO) treatment, or expressing p53^R175H^ oncogenic driver mutation. We observed that EECs and 3D-organoids with mtDNA depletion had cellular, morphological and genetic alterations typical of an oncogenic transition. Furthermore, mitochondrial dysfunction induced cellular transformation is accompanied by elevated mitochondrial fission protein, DRP1 and pharmacologic inhibition of mitochondrial fission by mDivi-1 in the *MPV17^-/-^* organoids reversed the phenotype to that of normal EEC organoids. Our studies show that mtDNA copy number depletion, activates a mitochondrial retrograde response, potentiates telomere defects, and increases the oncogenic susceptibility towards ESCC. Furthermore, mtDNA depletion driven cellular plasticity is mediated via altered mitochondrial fission-fusion dynamics.

## INTRODUCTION

Esophageal Squamous Cell Carcinoma (ESCC) is one of the most aggressive squamous cell cancers and is the sixth leading cause of cancer-related mortality in the world [1]. Esophageal cancer comprises of two main histological subtypes: esophageal squamous cell carcinoma (ESCC) and esophageal adenocarcinoma [2]. ESCC is the major histological type and accounts for >80% of esophageal cancer incidences worldwide [1]. Development of ESCC involves a multistep process that begins with a normal squamous epithelium and progression to low-grade intraepithelial neoplasia (LGIN), high-grade intraepithelial neoplasia (HGIN), and ultimately to invasive carcinoma [3]. Histopathologically, ESCC is defined on the basis of mitotic activity, nuclear atypia, and degree of squamous differentiation [3]. The most prevalent genetic alterations identified in ESCC include TP53^R175H^ or EGFR mutations [4]. Carcinogens such as 4-NitroQuinoline Oxide (4NQO) are reported to cause ESCC in mouse models [5]. ESCC is often diagnosed at advanced stage, accounting for its poor <20% survival rate further emphasizing the need to identify new markers for detection and therapeutic targeting.

Adaptation to the metabolic demands of proliferating cancer cells is critical to their survival, but is also their vulnerability. Mitochondria are cellular signaling hubs and integrate various metabolic pathways, synthesize intermediates required for the synthesis of biomass, maintain Ca^2+^ homeostasis and regulate apoptosis: cellular processes that are altered during oncogenic transformation. Functional interaction between mitochondria and nucleus controls both the biogenesis and functioning of mitochondria. Several cellular and environmental conditions disrupt mitochondrial function. Mitochondrial (mt) DNA mutations, deletions or impaired mtDNA replication are common causes of mitochondrial dysfunction. We have previously demonstrated that dysfunctional mitochondria alter the cytosolic Ca^2+^ pool and trigger a Ca^2+^-calcineurin dependent mitochondria-to-nucleus stress signaling pathway (MtRS) [6–11]. Activation of IGF1R, Akt and hnRNPA2 is essential for the propagation of MtRS [8–10]. It is established that mitochondrial dysfunction resulting from mutations in mtDNA, and nuclear DNA encoded genes for mitochondrial proteins, are associated with various types of tumors [12, 13]. However, the contribution of mtDNA copy number reduction in the initiation or progression of ESCC remains unclear.

To determine the role of dysfunctional mitochondria in ESCC progression, we utilized two different mouse models of mtDNA depletion: 1) *MPV17^-/-^* which contains tissue-specific mtDNA depletion [14, 15] and 2) *Tfam*^-/+^ mice [16, 17]. *MPV17* is a mitochondrial inner membrane protein. Loss of *MPV17* (*MPV17^-/-^*) causes mtDNA depletion and impairs oxidative phosphorylation (OXPHOS) in humans and mice, while the heterozygous (*MPV17*^+/-^) mice have mtDNA content and mitochondrial functions comparable to the wild type mice [14, 15]. *Tfam* is a mitochondrial transcription factor that controls mtDNA copy number. In most somatic tissues, *Tfam* levels correlate tightly with mtDNA content and *Tfam* heterozygous cells contain 50% reduced mtDNA while *Tfam* KO attain Rho^0^ state (total loss of mtDNA). These two models are therefore ideal to demonstrate the role of mtDNA defects and dysfunctional mitochondria in ESCCs. To study the ESCC oncogenic process *ex vivo*, we utilized the three-dimensional (3D) organoid system. 3D organoids provide a cell culture based, physiologically relevant, platform exhibiting tissue-like architecture grown in a mount of basement membrane extract with media containing the niche factors as described before [18]. The morphological and functional characteristics of a variety of tissues are recapitulated in 3D organoids generated from single-cell suspensions or cell aggregates isolated from murine and human tissues [19]. The 3D organoid system has emerged as a promising tool to study both normal development and homeostatic mechanisms as well as malignant transformation, to study alterations in genes and pathways associated with disease progression, gene-drug association, and design personalized therapy. Esophageal 3D organoids mimic normal esophagi as well as the tumorigenic transformation [18, 20].

We observed that primary cells and 3D organoids derived from esophageal epithelia of mice containing partial mitochondrial DNA (mtDNA) depletion shows the activation of the mitochondrial retrograde signaling (MtRS) pathway and cellular alterations that resemble oncogenic transition. In the *MPV17^-/-^* model of mtDNA depletion we observed telomere defects and chromosomal defects typical of tumor cells. Further, in the *MPV17^-/-^* organoids, we observed increased tumorigenic transformation and higher susceptibility to ESCC in response to oncogenic or carcinogenic stimuli. This is the first report that demonstrates the contribution of dysfunctional mitochondria towards 4NQO induced ESCC development using a novel murine mtDNA depletion 3D organoid model.

## RESULTS

### *MPV17* KO esophageal cells exhibit mtDNA depletion and cellular reprogramming

**Figure 1:**
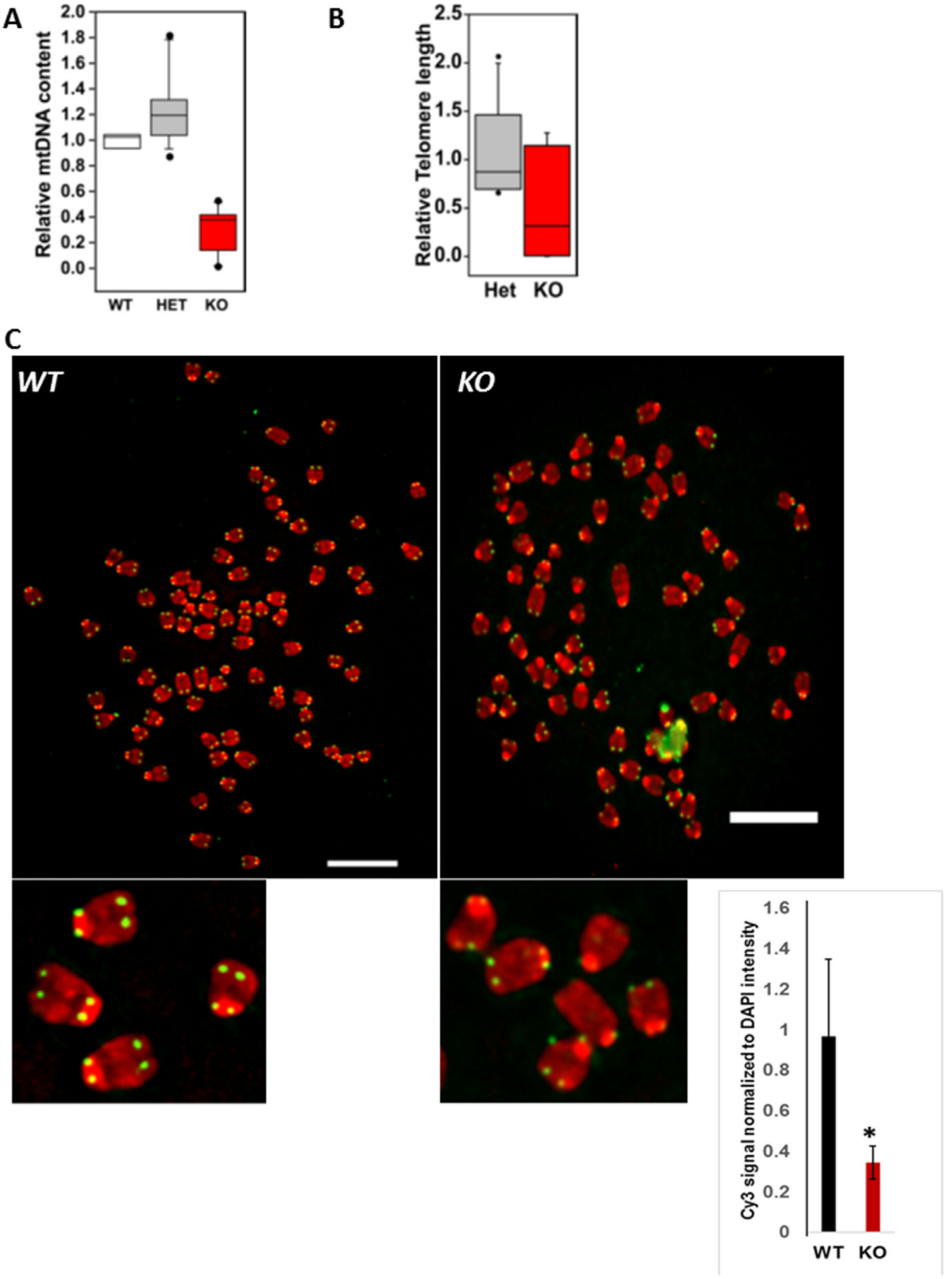
Primary esophageal epithelial cells derived from *MPV17*^**-/-**^ mice have mtDNA and telomere defects. (**A**) Relative quantitation of mtDNA (CcOI) in *WT and MPV17*^+/-^ *or MPV17^-/-^* EECs normalized to nuclear gene (CcOIVi1) analyzed by real time PCR. (**B**) Relative telomere length in *MPV17^-/-^* EECs compared to WT and *MPV*17^+/-^ analyzed by real time PCR. *N = WT* + *MPV17*^+/-^: *11; MPV17^-/-^*: *7; p < 0.05* (**C**) Representative image of telo-FISH of telomere Cy3-PNA probe (pseudo-colored in green) on metaphase spreads (pseudo-colored in red) in WT and *MPV*17*^-/-^* EECs. Inset shows metaphase and telomere signals. Scale bars indicate10 µm. Quantitation of telo-FISH metaphases (*n* = 10 per cell type). Significance *p* < 0.05 is indicated by ^*^.

We harvested the esophagi from either wild type mice (WT, *MPV17*^+/+^), *MPV17* heterozygotes (+/–) and homozygous knockout (–/–) mice (genotype shown in Supplementary Figure 1A). Single cells enzymatically dissociated from the mucosa were cultured *ex vivo* (referred henceforth as EEC) as described in Materials and Methods. In the *MPV17^-/-^* mouse model, the mtDNA depletion is tissue specific [14]. The EECs in *MPV17^-/-^* show 80% reduction in mtDNA content compared to WT (Figure 1A), whereas, the mtDNA content of EECs in *MPV17*^+/-^ mice is similar to WT mice.

A notable characteristic of cancer cells is shortened telomeres, and chromosomal abnormalities which increases the risk for chromosomal DNA damage and genomic instability. We recently reported that mtDNA depletion and mitochondrial dysfunction in immortalized cells activates mitochondrial retrograde signaling (MtRS) which plays a causal role in telomere attrition similar to that observed in tumor cells [21]. We therefore assessed the telomere length in primary esophageal epithelial cells (EEC) using real time PCR approach [21, 22] and observed that the median telomere length is markedly reduced in MPV17*^-/-^* esophageal tissues and esophageal cells compared to that of WT or *MPV17*^-/+^ (Figure 1B). Quantitative telomere fluorescent *in situ* hybridization (Tel-qFISH) analysis using telomeric DNA specific Cy-3 PNA probe showed marked loss of telomere signals (indicated by yellow arrows), higher telomeric signal-free ends at the chromatids and marked number of chromosome end-fusions in *MPV17^-/-^* EECs (Figure 1C). This suggests the association of mtDNA depletion with telomere defects (length attrition, fragile ends, end-fusions) which is consistent with our earlier observations in immortalized cells [21].

We further tested the contribution of mitochondrial stress in ESCC progression using human esophageal (epithelial) keratinocyte cell line EPC2-hTERT (EPC2) and EPC2-hTERT cells expressing the most prevalent ESCC mutation in gene TP53^R175H^ [23]. In agreement with our findings in primary EEC derived from *MPV17^-/-^* mice, human EPC2 cells [23] exhibit telomere attrition in response to mtDNA depletion and, the telomere loss correlates with the level of mtDNA depletion, suggesting a causal role of mtDNA depletion in telomere attrition (Supplementary Figure 2A).

### EECs derived from mtDNA depletion mouse models exhibit morphological alterations

The esophageal epithelial cells (EECs) from WT and *MPV17* *^+/-^* *or MPV17^-/-^* mice were harvested and enzymatically dissociated into single cells and grown either as two-dimensional cultures, or were suspended in Matrigel™ as detailed in Materials and Methods [20] to generate 3D organoids.

**Figure 2:**
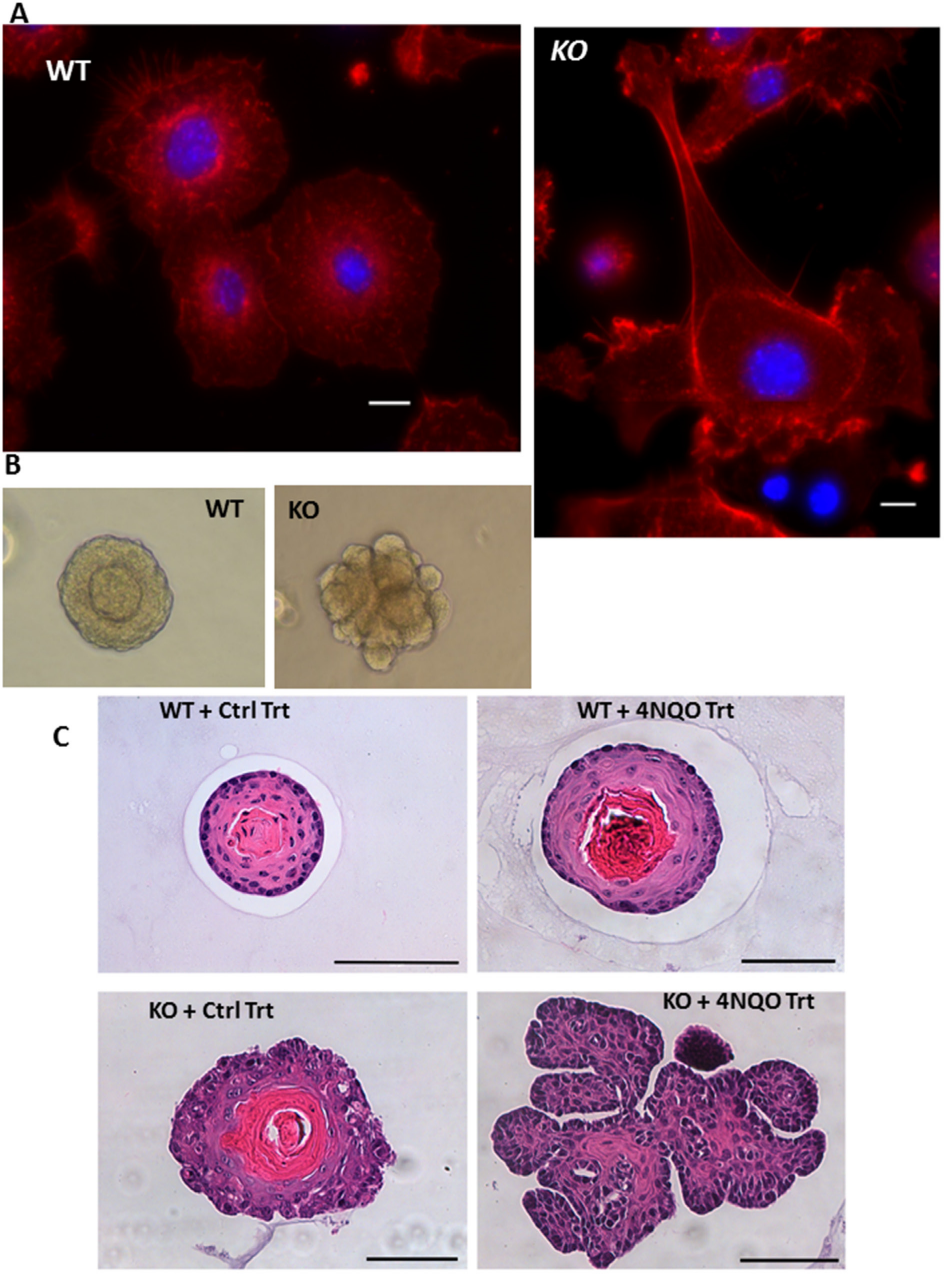
Altered morphology of *MPV17*^**-/-**^ organoids. (**A**) Phalloidin staining of F-actin (red) and nucleus (DAPI, blue) in WT or *MPV17^-/-^* EECs (as indicated) imaged under 100x objective in Leica wide field microscope. KO cells were imaged in multiple fields under same magnification and image tiles were stitched. Scale bar indicates 10 µm. (**B**) Representative bright field images of 3D organoids (Day 10) cultured from *WT* and *MPV17^-/-^* esophagi (**C**) Representative H&E stained sections of esophageal 3D organoids from *WT* and *MPV17^-/-^* mice treated with carcinogen 4NQO or control treatment as indicated in Materials and Methods.

Migration of cancer cells during metastatic transition is a complex and critical process requiring reorganization of actin filaments suggesting cytoskeletal remodeling. Prior studies have shown that actin and its interacting partners such as the Rho GTPases, along with downstream effector proteins mediate processes involved in tumor cell migration, invasion and metastasis through the cytoskeleton. Phalloidin staining of primary *MPV17^-/-^* EECs shows altered F-actin organization resulting in lamellipodia and filopodia membrane protrusion structures, and cells were markedly enlarged, typical of migratory cancer cells (Figure 2A). Phalloidin staining in mtDNA depleted human EPC2 cells shows actin reorganization and morphological alterations similar to tumor cells (Supplementary Figure 2B). Interestingly, EPC2 cells expressing oncogenic TP53^R175H^ mutation, exhibit similar morphological and actin reorganization as observed in mtDNA-depleted EPC2 cells suggesting that mtDNA depletion-induced mitochondrial stress mimics the oncogenic cellular transformation induced by tumor suppressor gene mutations in ESCC. Additionally, we observed similar morphological alterations in cytoskeletal actin reorganization in primary EECs derived from TFAM^+/fl^ +adeno CRE (Tfam^+/-^) mice (Supplementary Figure 1B and Supplementary Figure 3), which is our parallel model for mtDNA depletion showing the characteristic mtDNA content reduction and telomere attrition (Supplementary Figure 4A–4C).

The 3D organoids generated from WT mice were morphologically similar to normal esophageal tissue after 10 days in culture containing small basal-like cells in contact with the extracellular matrix, large flat suprabasal-like cells in the interior and a keratinized region in the center. In contrast organoids formed from MPV17*^-/-^* mice formed dysmorphic structures invading into the surrounding Matrigel™ matrix typical of oncogenic phenotype (Figure 2B, 2C).

We observed that *MPV17* *^-/-^* animals did not develop any tumors *in vivo* and there are no reports from other laboratories of spontaneous tumors in *MPV17^-/-^* mice. Therefore, we tested the possibility that mitochondrial dysfunction in *MPV17^-/-^* mice increases the tumorigenic potential but requires additional oncogenic challenge to form tumors *in vivo*. Based on this rationale, we treated *MPV17^-/-^* mice with a potent esophageal carcinogen, 4 Nitroquinoline-N-Oxide (4NQO) for 8 weeks. This treatment duration is substantially shorter compared to the 16 weeks of 4NQO treatment followed by 12 weeks of observation used in other studies for inducing esophageal cancers. We observed that *MPV17^-/-^* animals exhibited loss of body weight (nearly 20%) while there was no significant weight loss in either the WT or *MPV17*^-/+^ mice suggesting that 4NQO treatment had substantially higher deleterious effects on the *MPV17^-/-^* mice (Supplementary Figure 5A). There were visible lesions in the esophagus of the *MPV17^-/-^* mice while no abnormality was observed in esophagus in WT or *MPV17*^-/+^ mice. Histopathological analysis of the esophageal sections indicated pre-cancerous lesions including esophageal hyperplasia and dysplasia in *MPV17^-/-^* mice without any abnormality in esophagi of WT mice. Mild hyperplasia with basal layer activity and some minor dysplasia indicated by a mild cell polarization alteration, some hyperchromatic nuclei and mild changes in nuclear size and shape were observed in *MPV17^-/-^* mouse esophagi (Supplementary Figure 5B). Additionally, we observed basal/parabasal cell vacuolization in some areas consistent with observed effects of carcinogens (Supplementary Figure 5B).

Figure 2C represents 3D organoids from esophageal epithelial cells from the WT and *MPV17^-/-^* mice treated with 4NQO as described above. H&E staining of the organoid cross sections demonstrate that *MPV17^-/-^* organoids have dysplastic mucosa consistent with neoplastic transformation (Figure 2C). Similar to our previous study with mtDNA depleted immortalized cells, the TFAM^+/fl^ +adeno CRE (*Tfam*^+/-^) primary EECs were larger in size (Supplementary Figure 6A) and had reduced mitochondrial membrane potential (Supplementary Figure 6B).

### Oncogenic and carcinogenic stimuli potentiate MtRS activation and cellular plasticity in MPV17*^-/-^* organoids

**Figure 3:**
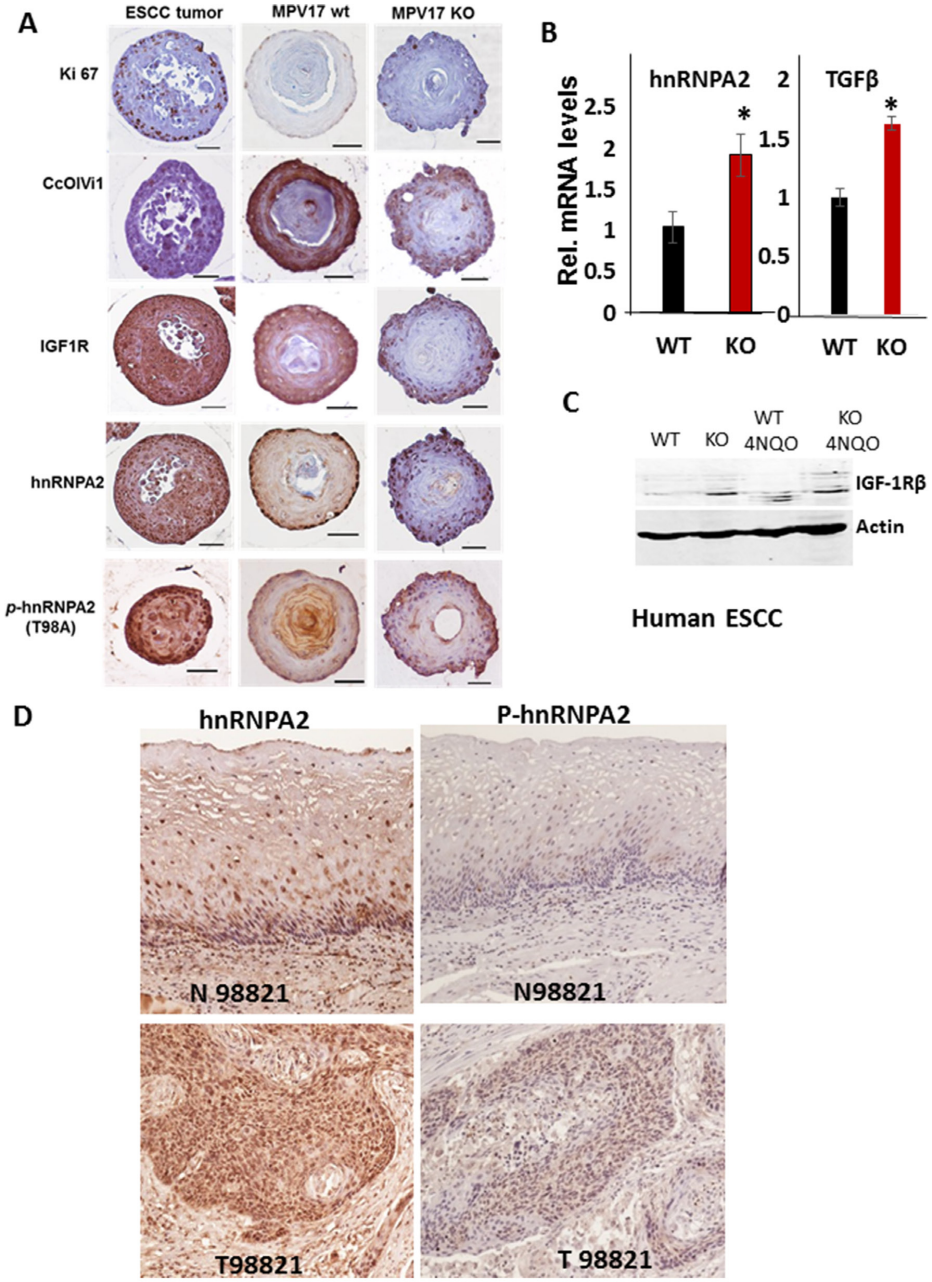
*MPV17^-/-^* esophageal 3D organoids show elevated expression of oncogenic MtRS marker proteins. (**A**) Representative images of immunohistochemical analyses of parallel sections of esophageal organoids from *WT and MPV17^-/-^* mice treated with 4NQO and stained for Ki-67, CcOIVi1, IGF-1R, hnRNPA2 and p-hnRNPA2. Images were captured on Leica wide-field microscope under 40x objective. Pathologically verified mouse ESCC tumor sections were used as positive control for antibody staining. Scale bar 50 µm. (**B**) Real Time PCR analyses showing the mRNA levels of MtRS marker genes, hnRNPA2 and TGFβ in WT and *MPV17^-/-^* EEC. (**C**) Western immunoblot showing the protein levels of MtRS marker gene IGF1R in *WT and MPV17^-/-^* EEC treated with either 4NQO or the control. (**D**) Representative human ESCC tumor sections stained for MtRS markers hnRNPA2 and p-hnRNPA2.

We next analyzed the 3D organoids derived from esophagi of 4NQO treated WT and *MPV17^-/-^* mice (Figure 3A). Compared to WT organoids, *MPV17^-/-^* mouse derived organoids showed higher expression of Ki-67 (intense staining) similar to esophageal tumors indicative of high proliferation as observed in neoplastic cells. The mitochondrial retrograde signaling (MtRS) pathway can be triggered by mtDNA depletion or by loss of nuclear encoded mitochondrial electron transport chain protein CcOIVi1, [7–11, 24–26]. Activation of hnRNPA2 and activated phospho-hnRNPA2 (T98A) are critical mediators of this signaling pathway [10, 27]. In response to 4NQO treatment, we observed increased levels of MtRS factors IGF-1R, hnRNPA2, phospho-hnRNPA2 and reduced CcOIVi1 in *MPV17^-/-^* organoids compared to *WT* organoids (Figure 3A).

We further assessed the presence of MtRS signaling pathway and oncogenic potential in primary *WT or MPV17^-/-^* EECs. The mRNA levels for the MtRS markers, hnRNPA2 and TGFβ and IGF1R protein level are higher in *MPV17^-/-^* EEC compared to WT (Figure 3B and 3C) suggesting that mitochondrial stress potentiates the effect of nuclear oncogenic mutations in eliciting an oncogenic response. We analyzed human esophageal squamous carcinoma (ESCC) sections for hnRNPA2 induction and activation and observed that hnRNPA2 and phospho-hnRNPA2 (hnRNPA2 activation) levels were elevated in tumor ESCC sections compared to matched normal tissues demonstrating the induction of MtRS in esophageal tumors (Figure 3D).

**Figure 4:**
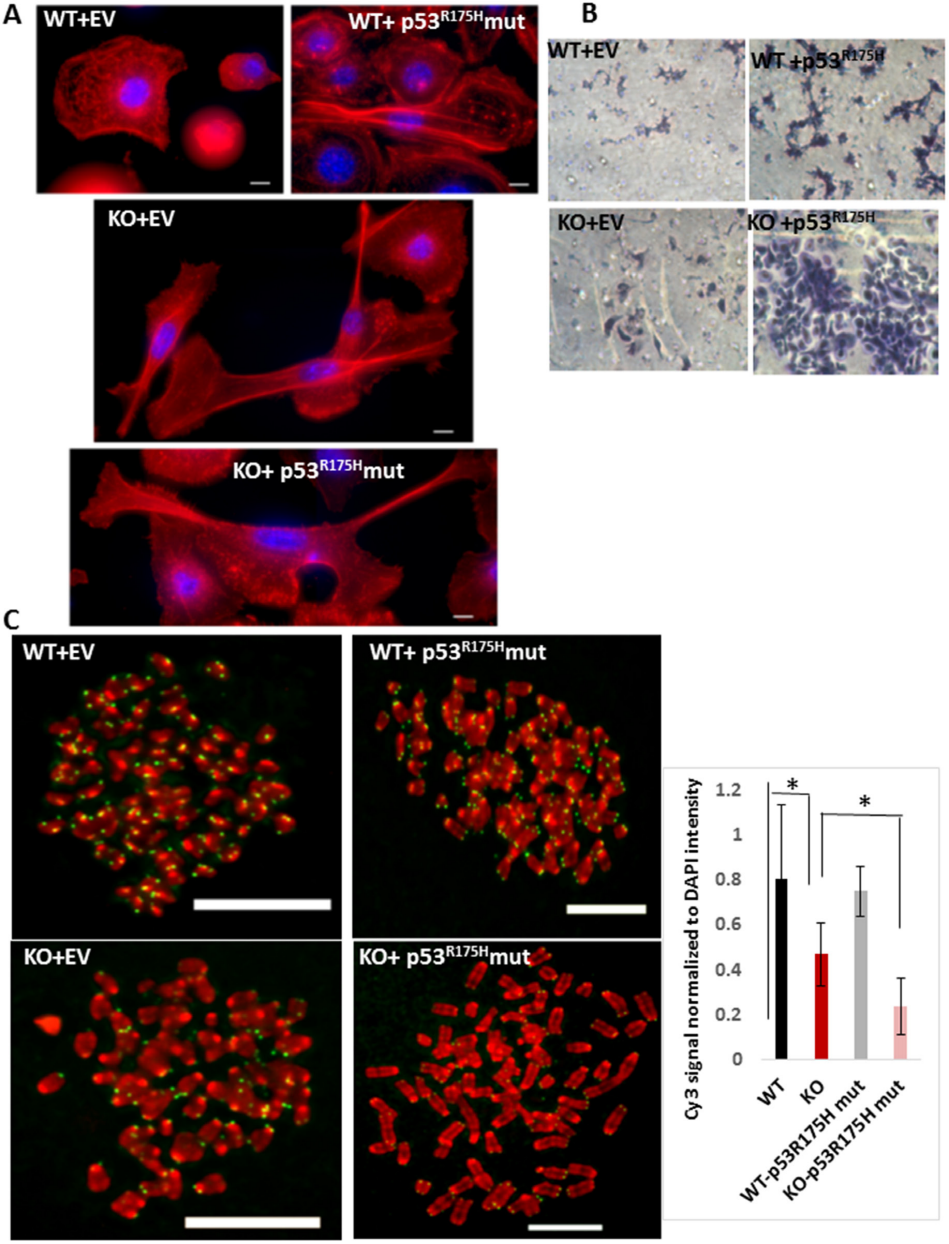
(**A**) Phalloidin staining of F-actin (red) and nucleus (DAPI, blue) in WT or *MPV17^-/-^* EECs expressing the empty adenoviral vector (control) or the TP53^R175H^ mutant (as indicated) imaged under 100x objective in Leica wide field microscope. KO cells were imaged in multiple fields under same magnification and image tiles were stitched. (**B**) Representative bright field images showing the *in vitro* Matrigel invasion in *WT and MPV17^-/-^* EEC expressing either the empty adenoviral vector (control) or the TP53^R175H^ mutant. (**C**) Left: Representative images of telo-FISH of telomere Cy3-PNA probe (pseudo-colored in green) on metaphase spreads (pseudo-colored in green) in WT and *MPV*17*^-/-^* EECs expressing the empty adenoviral vector (control) or the TP53^R175H^ mutant (as indicated) imaged under 100x objective in Leica wide field microscope. Scale bars indicate 10 µm. Right: Quantitation of the telo-FISH metaphases (*n* = 5 to 10 per cell type). Significance *p* < 0.05 is indicated by ^*^.

Because we observed actin reorganization in *MPV17^-/-^* EECs in an earlier experiment (Figure 1C), we investigated the effect of oncogenic TP53^R175H^ mutation in these cells (Figure 4A). As expected, WT EECs expressing the oncogenic TP53^R175H^ mutation showed remodeling of actin filaments exhibiting filopodia typically observed in cancer cells. WT EECs expressing the vector control resembled normal EECs. Notably, both the *MPV17^-/-^* and *MPV17^-/-^*+ TP53^R175H^ EECs undergo actin reorganization similar to migratory cancer cells (Figure 4A). Importantly, actin reorganization observed in *MPV17^-/-^* cells is similar to WT cells expressing the oncogenic TP53^R175H^ mutation, which further suggests that mtDNA depletion can mimic oncogenic stimuli in inducing cellular reprogramming.

Based on our results above suggesting an oncogenic potential in *MPV17^-/-^* EECs, we assessed their invasive potential by an *in vitro* Matrigel™ invasion assay which recapitulates the capacity of cells to invade through a Matrigel™ layer enriched with components of the tumor microenvironment. As expected, WT EECs expressing the empty vector (control) were non-invasive while WT cells expressing the oncogenic TP53^R175H^ mutation acquired invasive capacity (Figure 4B, top panel). While the *MPV17^-/-^* cells showed marginal invasive potential, *MPV17^-/-^*+ TP53^R175H^ EECs demonstrated robust invasiveness (Figure 4B, bottom panel).

Furthermore, our Telo-FISH results in Figure 4C show that compared to WT mice, which showed normal telomeres, *MPV17*^-/-^+ TP53^R175H^ primary EECs showed no significant reduction in telomere signals. Telomeres in *MPV17^-/-^* EECs expressing the adenoviral vector as control showed significant loss (~50% reduction) in telomere signals similar to our earlier observation in Figure 1C. *MPV17^-/-^*+ TP53^R175H^ EECs show a further 45% reduction compared to *MPV17^-/-^* EECs and 75% loss in telomere signals compared to that in WT EECs (Figure 4C). This shows that mtDNA reduction combined with a nuclear oncogenic mutation induces severe telomere loss, which potentially can cause chromosomal aberrations frequently observed in ESCCs.

We observed a similar pattern of invasiveness in the Tfam^+/-^ EECs, which is our alternate mtDNA depletion model. Tfam^+/-^ EECs immortalized using T-antigen showed markedly higher invasive potential (Supplementary Figure 4D). Our results suggest that mitochondrial stress provides an oncogenic stimulus, which augments the effects of oncogenic mutations to induce transition to cancer phenotype.

### Cellular plasticity in *MPV17^-/-^* 3D organoids is associated with altered mitochondrial dynamics

**Figure 5:**
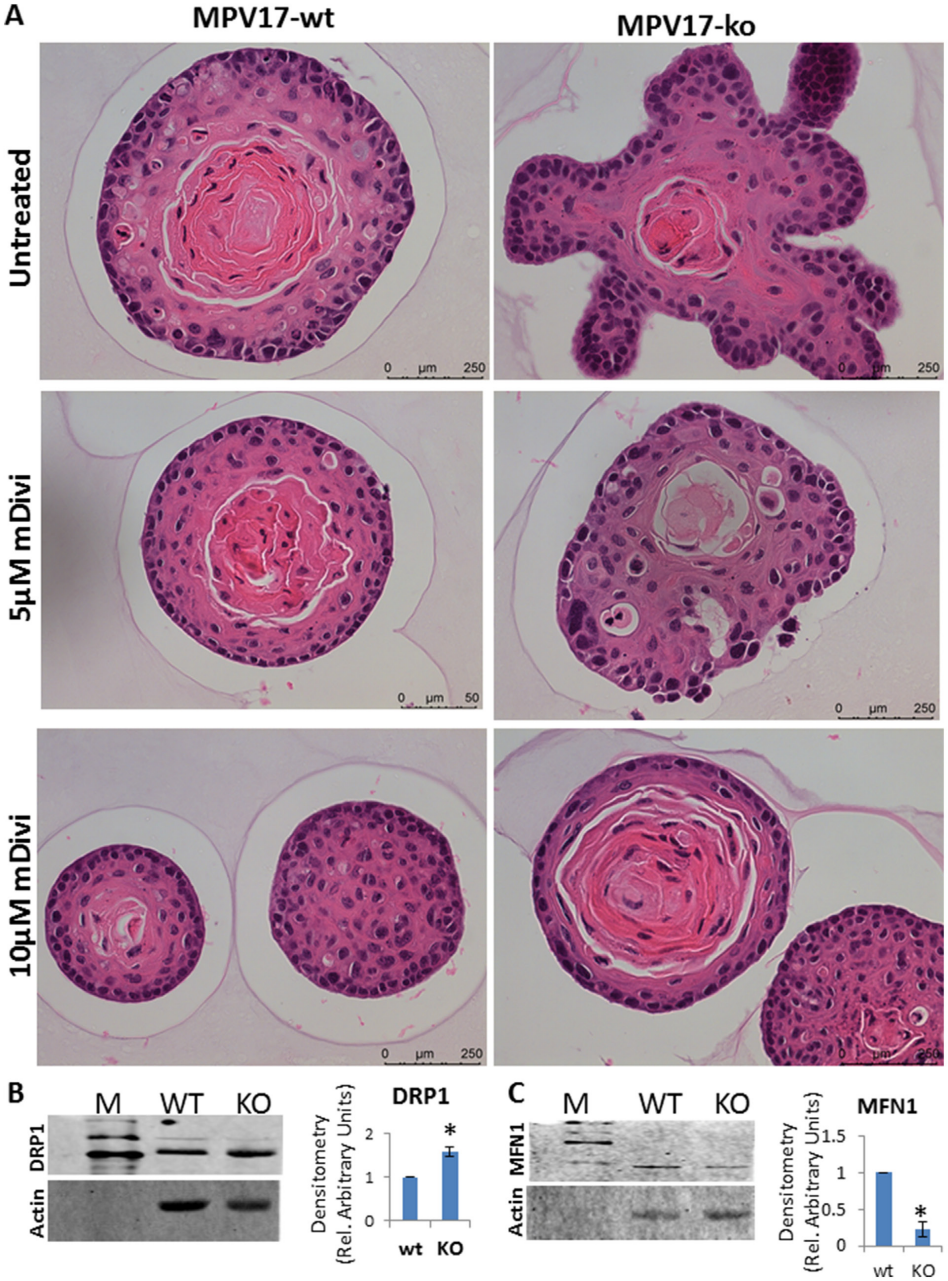
(**A**) Representative bright field images of H&E stained *WT and MPV17^-/-^* esophageal organoids treated with DRP1 inhibitor mDivi-1 as indicated in the figure. Treatment was started at day 5 of the organoid culture. (**B**, **C**, left panel) Representative western immunoblot from 50 µg total cell lysates from WT and *MPV17*17*^-/-^* EECs showing protein levels of mitochondrial (**B**) fission marker protein DRP1 and (**C**) fusion marker protein MFN1. (**B**, **C** right panel) Densitometry analysis from two replicate blots. ^*^ *P* < 0.05.

Several groups have reported elevated mitochondrial fission and higher DRP1 in response to mitochondrial stress [26, 28–30]. During neoplastic transformation, large-scale genomic and proteomic analyses have reported a correlation between Dynamin related protein-1(DRP-1) and cell cycle genes in 29 different cancer cell types, in which DRP1 reportedly drives mitotic transition [31]. Hence, we tested the hypothesis that increased mitochondrial fission was a critical adaptation to altered bioenergetic requirements during cellular transition we observed in *MPV17^-/-^* organoids. Therefore, we treated the organoids *ex vivo* with DRP1 inhibitor, mDivi-1 to inhibit mitochondrial fission and observed a reversal in the *MPV17^-/-^* organoid morphology towards the normal phenotype suggestive of the involvement of higher fission in driving the cellular plasticity in this model of mitochondrial dysfunction (Figure 5A). Notably, reports show that inhibition of fission inhibits cancer progression [28, 29, 32, 33]. In *MPV17^-/-^* EECs, we observed higher levels of the fission protein DRP1 (Figure 5B). Mitochondrial fusion, which is the opposing process to fission, is reduced in tumor cells [34]. In agreement, *MPV17^-/-^* cells have reduced protein levels of the mitochondrial fusion marker, MFN1 (Figure 5C).

## DISCUSSION

Increasing number of reports suggest that mitochondrial dysfunction is associated with initiation or progression of cancers. Some cancers, on the other hand, seem to require a robust mitochondrial function for propagation and tumors with high levels of OXPHOS and high mtDNA contents invariably reflect the resistance of tumors against cancer therapies [35]. Mitochondrial genome defects have been associated with various aggressive cancers. ESCCs are clinically challenging because they are diagnosed at an advanced stage thereby limiting the available treatment options. Reports show that mtDNA copy number changes including high or low, mtDNA mutations and deletions in tumor tissues [36]. In fact, frequent mtDNA mutations in the D-loop 4977 bp, a common deletion, was detected in 92% of esophageal tumors [37] and as high as 182 mutations in the protein coding genes have been reported in human esophageal cancers [38–40]. These findings suggest the possibility that mtDNA defects and mitochondrial dysfunction potentially contribute to the etiology of ESCCs. Our prior studies have shown that mtDNA copy number depletion and dysfunctional mitochondria activate mitochondria to nucleus retrograde signaling (MtRS) pathway, which reprograms immortalized skeletal myoblasts into tumorigenic phenotype [7, 9, 11, 24] as well as contributes to the metastatic phenotype of cancers of the mammary, lung and osteosarcomas [24, 25, 41, 42]. In this study, we demonstrate that mtDNA copy number depletion in murine primary esophageal epithelial cells can activate MtRS and result in oncogenic transformation. Furthermore, our results suggest that mitochondrial stress potentiates the effect of carcinogens and oncogenic nuclear DNA mutations.

Three-dimensional organoids have gained importance as study models because they recapitulate the tissue development, heterogeneity and disease progression phenotype in a shorter time and can therefore be utilized for understanding the underlying mechanisms, drug screening and for designing personalized therapeutics [43]. In this study, we generated novel murine 3D esophageal organoid model of mtDNA depletion and demonstrated that mtDNA depletion influences cellular reprograming including telomere attrition, marker gene expression, filopodia, invadopodia formation and invasiveness, typical of ESCC oncogenic transformation.

Mitochondrial morphology is influenced by proteins that control the dynamic equilibrium between fusion and fission of the mitochondrial network. Their balance is strictly required to regulate various processes, including the quality of mitochondria, cell metabolism, cell death, proliferation and cell migration. Healthy mitochondria are dynamic and morphologically fluid which facilitate both the efficiency of mitochondrial function and turnover. Alterations in these processes are frequently encountered in cancer, during both initiation and progression and alterations in mitochondrial dynamics is associated with cancer development. The mitochondrial morphology is the result of the interplay between rapid fusion and fission events and is brought about by GTPases Mitofusin (MFN1) and Dynamin-related-protein 1 (DRP1) [28]. Our results here showing that inhibition of mitochondrial fission by mDivi-1 reversed the cellular phenotype of the *MPV17^-/-^* cells, is in agreement with the dogma that increased mitochondrial fission is an adaptive mechanism of cancer cells [28] and that pharmacologic inhibition of fission by mDivi-1 may prove therapeutically beneficial in ESCC patients.

Epidemiological studies suggest an association with tobacco and alcohol abuse [44]. Additionally 4-Nitroquinoline-N-oxide (4NQO), a water-soluble carcinogen develops squamous cell carcinoma after 8–16 weeks of 4NQO treatment followed by 16 weeks of no treatment [5]. We observed preneoplastic transformations in the *MPV17^-/-^* esophageal epithelium in less than 8 weeks. This shorter treatment duration required to induce carcinogenic alteration in *MPV17^-/-^* compared to the WT or *MPV17*^-/+^ mice suggests that mitochondrial dysfunction is an additive factor in potentiating the carcinogenic effect of 4NQO. Furthermore, we observed that in human esophageal keratinocytes, chemically induced-mtDNA depletion resulted in loss of telomere length typical of tumor cells. This is in agreement with our recent report showing that mitochondrial stress plays a causal role in telomere attrition and chromosomal aberrations in skeletal myocytes [21]. Other reports also suggest the contribution of dysfunctional mitochondria to telomere defects [21]. A study demonstrated that mtDNA haplotype influences mitochondrial dysfunction and associated aging parameters such as telomere attrition in conplastic mice [22].

Based on our data here, we propose that while mitochondrial dysfunction by itself may not be sufficient for ESCC initiation, the presence of carcinogenic and/or nuclear oncogenic stimulus increases ESCC susceptibility and can potentially dictate the tumorigenic progression and therapy outcome. We earlier reported that cigarette smoke toxins and environmental carcinogens have deleterious effects on mitochondrial functions [45, 46]. Individuals exhibiting mitochondrial defects when exposed to environmental carcinogens may be at higher risk of developing aggressive ESCC. Therefore, identifying new environmental and genetic factors and molecular biomarkers are important in stratifying patients who are at risk for aggressive disease as well as for designing personalized therapy. Furthermore, our results suggest pharmacologic intervention of mitochondrial fission can be therapeutically beneficial in ESCC patients. This study demonstrates the contribution of reduced mtDNA content induced mitochondrial dysfunction towards an oncogenic transformation in ESCCs and opens up a new mitochondria-based approach for early detection and therapy in the future.

## MATERIALS AND METHODS

### Animals

The MPV17 knock out mice used in this study were obtained from Jackson Laboratories (CFW-MPV17/J, JAX stock #002208) and bred to BALB/c mice for 10 generations before using for experiments. At the initiation of this study, all animals used were age matched (8 weeks) and represented both sexes. Animals were housed and cared for in accordance with the regulations of the University of Pennsylvania’s Institutional Animal Care and Use Committee. Mice were euthanized using CO2 asphyxiation using an IACUC approved protocol before harvesting tissues.

The genotyping primers used are as follows:

MPV1717 WT/FW AACCACTACGGCTGGCTAGA.

MPV1717 WT/RC GCTTCAAAGCAAACGACCTC.

MPV1717 MUT/RC CCTACAGGTGGGGTCTTTCA.

### Primary 2D esophageal epithelial cells

All tissue isolation from the MPV17 and Tfam mice were performed according the approved IACUC protocol (Protocol Number: 805731). Primary murine esophageal epithelial cells were harvested following standard protocol [20]. Briefly, tissues were placed in Hanks BSS buffer, transferred to dispase (0.6 ul/ml in PBS), then trypsinized (0.05% trypsin-EDTA) for 20 minutes at 37°C. Trypsin was inactivated using soybean trypsin inhibitor (Sigma), and tissues were agitated at 37°C for 30 minutes in a shaker-incubator to release the epithelial cells. Cells were pelleted, resuspended, and seeded. While cells after seeding in the initial passage were heterogeneous, by passage 3, the cell population was homogeneous and exhibited markers of primary esophageal epithelial cells. Primary EECs were cultured in keratinocyte serum-free media (Invitrogen) supplemented with bovine pituitary extract (50 μg/ml), epidermal growth factor (1 ng/ml), Amphotericin B and penicillin/streptomycin (100 units/ml), at 37°C in a humidified 5% CO2 incubator. Cell line identity was verified using mRNA levels of the genes (*mpv17* and *tfam*) as well as by genomic profiling.

Cell size and viability was measured by staining cells suspensions (20 μl) with 0.2% trypan blue (Sigma, T8154) and analyzed using an automated cell counter (Cellometer Vision, software version 2.1.4.2, Nexcelom Biosciences, Lawrence MA). Identical number of cells (between 1000–1500) were counted for each cell type.

### *Ex vivo* 3D organoid culture

Esophageal keratinocytes were isolated from untreated, control treated or 4NQO- treated mice. Using 24-well plates, 5000 cells were seeded per well in 50 μl Matrigel. After solidification, 500 μl of DMEM/F12 supplemented with 1× Glutamax, 1× HEPES, 1× N2 Supplement, 1× B27 Supplement, 0.1 mM N-acetyl-cysteine (Sigma-Aldrich), 50 ng/ml mouse recombinant EGF (R&D Systems), 2.0% Noggin/R-Spondin-conditioned media and 10 μM Y-27632 (Tocris Biosciences, Bristol, UK) were added and replenished every other day. Organoid formation rate was calculated as the percentage of the number of organoids formed at day 7 per total number of cells seeded at day 0. After 10 days, the organoids were recovered by digesting Matrigel with Dispase I (BD Biosciences; 1 U/ml) and fixed overnight in 4.0% paraformaldehyde. Specimens were embedded in 2.0% Bacto Agar and 2.5% gelatin prior to paraffin embedding. Cross sections (50 µm) of the organoids were stained with hematoxylin-eosin. Immunohistochemistry using antibodies (as indicated) was performed on parallel sections.

### Quantification of mtDNA

Analysis of MtDNA content from cellular total DNA was performed using real time qPCR as described before [41]. Copy number of MtDNA coded gene (CcO I) was normalized to nuclear single copy gene (CcO IVi1).

### Primer sequences:

COX1 gene:

TGATCTGCTGCAGTGCTCTGA (forward).

TCAGGCCACCTACGGTGAA (reverse).

COX IV1i gene:

GAAAGTGTTGTGAAGAGCGAAGAC (forward).

GTGGTCACGCCGATCCAT (reverse).

### Quantitative real time PCR

Total cellular RNA was prepared using the RNeasy mini kit™ (Qiagen Cat # 74104). Genomic DNA was eliminated from the RNA preparations using Turbo DNA Free kit™ (Thermo Fisher Scientific). 1 μg RNA was reverse transcribed into cDNA using High Capacity reverse transcription kit (Applied Biosystems). 100 ng cDNA was used for each SYBR Green reaction for transcript analysis of the genes as indicated. Quantitative Real Time PCR assays were run on an ABI Quant Studio 6 real time thermocycler (Applied Biosystems). The nuclear gene actin was used as an endogenous control. All real time PCR assays were run in triplicate. Data are presented as Relative Quantification (RQ).

### Invasion assay

*In vitro* invasion assays were performed as described before [27, 47]. Cells (5 × 10^4^) in growth medium were seeded on a Matrigel-coated Boyden chamber. After 24 h, cells that invaded the Matrigel were stained with hematoxylin-eosin and observed under bright field microscope.

### Immunohistochemistry

Immunostaining of human esophageal cancer tissue or mouse esophageal organoid sections was done using Vectastain ABC kit (Vector laboratories, Burlingame, CA, USA) according to manufacturer’s instructions. Briefly, sections were de-paraffinized and incubated in blocking buffer for 1 h at 37°C. Incubation with primary antibodies was carried out overnight at 4°C. Biotinylated secondary IgG incubation was then carried out at 37°C and the signal was developed using the DAP peroxidase staining kit (Vector laboratories).

### Immunocytochemistry

Cells (2 × 10^5^ cells per well on a 6-well plate) were grown overnight in growth medium on Poly-D Lysine coverslips. Cell adherence and confluence was confirmed before processing. After 24 h of cell seeding, cells were washed with 1X PBS and fixed in ice-cold methanol for 10 minutes, at room temperature. Fixed cells were blocked in a buffer containing 1% BSA and were incubated with primary antibodies for 1 hour at 37°C. Immunostained cells were imaged using a Leica confocal microscope under a 100x objective.

### Metaphase chromosome preparation, Q-FISH and image analysis

Cells were treated with 0.1 µg/ml Colcemid solution (Sigma, St. Louis, MO) for 3 hours and harvested for metaphase spreads. Cells were swollen in hypotonic solution at 37°C for 15 minutes, and fixed with methanol: acetic acid (3:1) with three repeated exchanges prior to dropping onto slides and dried overnight.

Nuclei and metaphase spreads were processed for telomere Q-FISH. After washing and hypotonic swelling, cells were fixed and stored in methanol/acetic acid fixative using standard procedures [21]. Nuclei and metaphase spreads were fixed on slides. The slides were dried overnight in air and immersed in PBS for 5 min prior to fixation in 4% formaldehyde in PBS for 2 min; slides were then washed in PBS (3 × 5 min) and treated with pepsin (Sigma, St. Louis, MO) at 1 mg/ml for 10 min at 37°C, pH 2.0. After a brief rinse in PBS, the formaldehyde fixation and washes were repeated and the slides were dehydrated with ethanol and air-dried. Hybridization mixture containing 70% formamide, Cy3 PNA probe (Cy3-OO-CCCTAACCCTAACCCTAA), and 1% (W/V) blocking reagent in 10 mM Tris pH 7.2 was added to the slide, a coverslip (20 × 20 mm) was added and DNA was heat denatured. After hybridization for 2h at room temperature, the slides were washed at room temperature with 70% formamide/10 mM Tris pH 7.2 (2 × 15 min) and with 0.05 M Tris 0.15 M NaCl pH 7.5 containing 0.05% Tween-20 (3 × 5 min). The slides were then dehydrated with ethanol, air-dried and covered with Aquamount solution (Thermo Scientific, Philadelphia PA) containing 0.1 µg/ml of DAPI.

The nuclei and metaphases on the PNA hybridized slides were visualized under a Nikon microscope and images were captured under 100x objective. The image acquisition conditions were kept identical for all cell types. For quantitation, the raw images of nuclei were used for analysis using MetaMorph software (Molecular Devices). Cy3-PNA signals were counted and the fluorescence intensity was quantitated by applying consistent intensity and size thresholds. The average DAPI fluorescence intensity for each nucleus was quantified and used to normalize the measured Cy3 PNA fluorescence intensities. The total DAPI fluorescence signal for each nucleus was quantified. At least 10 nuclei were counted for each cell type.

Telomere-FISH: Cy3 PNA probe: Cy3-OO-CCCTAA CCCTAACCCTAA.

### Statistical analysis

All experiments were performed in biologic and technical replicates. Data are presented as means ± SEM and statistical significance was determined using a 2-tailed unpaired Student *t* test. *P* value of < .05 was considered statistically significant. Statistical analyses were performed using Prism software (GraphPad, La Jolla, CA).

## SUPPLEMENTARY MATERIALS


