# Supplementary material for: Esophageal 3D organoids of MPV17-/- mouse model of mitochondrial DNA depletion show epithelial cell plasticity and telomere attrition
Source: Oncotarget. 2019 Oct 22;10(58):6245–59. doi: 10.18632/oncotarget.27264 (PMC6817447; doi:10.18632/oncotarget.27264)
Supplement: Supplementary file 1 [file oncotarget-10-6245-s001.pdf]

Esophageal 3D organoids of *MPV17*$^{-/-}$ mouse model of mitochondrial DNA depletion show epithelial cell plasticity and telomere attrition

SUPPLEMENTARY MATERIALS

MATERIALS AND METHODS

TFAM KO animals

Tfam expression is allelic copy number dependent (heterogyzous KO have 50% reduced and homogyzous KO have 100% reduced Tfam mRNA compared to wt). Esophageal epithelial cells isolated from TFAM$^{+/fl}$ mice were transduced *ex vivo* with either adenoCMV or adenoCRE. mtDNA copy number correlates with the Tfam transcript levels.

The genotyping primers used are as follows:

TFAM A: CTGCCTTCCTCTAGCCCGGG.

TFAM B: GTAACAGCAGACAACTTGT.

TFAM C: CTCTGAAGCACATGGTCAAT.

Expected size of PCR products: WT: 400 kb; KO: 320.

Cell culture

Esophageal keratinocytes (EPC)

Primary human esophageal keratinocytes, designated as EPC2, were established as described previously [1]. Cells were maintained at 37°C and 5% CO_2 using keratinocyte-SFM medium (KSFM; Invitrogen) supplemented with 40 μg/mL bovine pituitary extract (Invitrogen), 1.0 ng/mL EGF (Invitrogen), 100 U/mL penicillin, and 100 μg/mL streptomycin (Invitrogen). EPC2 cells expressing EPC2–hTERT and EPC2–hTERT–p53R175H were have been described earlier [2].

Tfam floxed esophageal epithelial cells

For *ex vivo* recombination experiments, esophageal epithelial cells isolated from Tfam$^{+/fl}$ mice were transduced with adenovirus vector containing either CMV or CRE recombinase (University of Iowa Gene Transfer Vector Core). Adenovirus vector containing CMV is used as control. Adenovirus vectors were used at 1:500.

4Nitroquinoline- N-Oxide (4NQO) treatment *in vivo*

*MPV*17$^{-/-}$ mice (*n* =), 7.0 weeks age received 100 μg/ml 4NQO in 2% propylene glycol (MP Biomedicals, Solon, OH) in drinking water ad libitum for 8 weeks (or earlier if mice developed visible signs of discomfort or sickness) and followed for another 4 weeks without treatment. In parallel experiment, littermates were treated with 2% propylene glycol as control group. All experiments and treatments were done under approved protocol from the University of Pennsylvania Institutional Animal Care and Use Committee (IACUC #805731). Both male and female mice were included in this study and animals were randomized to treatment groups.

Histopathological examination

At necropsy, the entire length of the esophagus was dissected longitudinally. The tongue and the esophagus were examined for the presence of macroscopic topographical alterations. The entire esophagus were fixed overnight in 10% neutral buffered formalin, transferred to 70% ethanol and embedded in paraffin. 5 µm thick sections were obtained from the paraffin-embedded blocks and stained with hematoxylin and eosin. Sections were examined for the presence of epithelial atypia, dysplasia and neoplasia using established criteria. Normal histopathology was assessed by examining sections from the wild-type, non-carcinogen treated mice. Subsequent sections were analyzed blinded to the genotype and carcinogen treatment. Depending on the degree of atypical cytological and morphological changes, lesions were scored as mild, moderate or severe hyperplasia and dysplasia.

Mitochondrial membrane potential

Mitochondrial membrane potential in primary EECs derived from TFAM$^{+/fl}$ + adenoCMV and TFAM$^{+/fl}$ + adenoCRE was analyzed using the Cellometer Vision CBA. The MitoProbe™JC-1 (Invitrogen, Carlsbad, CA) assay was used for mitochondrial membrane potential analysis. The assay measures the depolarization of mitochondrial membrane potential. Cells were stained by the JC-1 dye, which exhibits membrane potential-dependent accumulation in mitochondria. The JC-1 fluorescent dye forms red fluorescent J-aggregates (590 nm) when accumulated in the mitochondria. Depolarization of mitochondrial membrane potential was indicated by JC-1 emission shifting from red (590 nm) to green (529 nm) fluorescence (indicated by lower FL2 intensity) due to the loss of concentration-dependent formation of red fluorescent J-aggregates.

REFERENCES

1. Andl CD, Mizushima T, Nakagawa H, Oyama K, Harada H, Chruma K, Herlyn M, Rustgi AK. Epidermal growth factor receptor mediates increased cell proliferation, migration, and aggregation in esophageal keratinocytes *in vitro* and *in vivo*. J Biol Chem. 2003; 278:1824–30. https://doi.org/10.1074/jbc.M209148200. [PubMed]

2. Okawa T, Michaylira CZ, Kalabis J, Stairs DB, Nakagawa H, Andl CD, Johnstone CN, Klein-Szanto AJ, El-Deiry WS, Cukierman E, Herlyn M, Rustgi AK. The functional interplay between EGFR overexpression, hTERT activation, and p53 mutation in esophageal epithelial cells with activation of stromal fibroblasts induces tumor development, invasion, and differentiation. Genes Dev. 2007; 21:2788–803. https://doi.org/10.1101/gad.1544507. [PubMed]

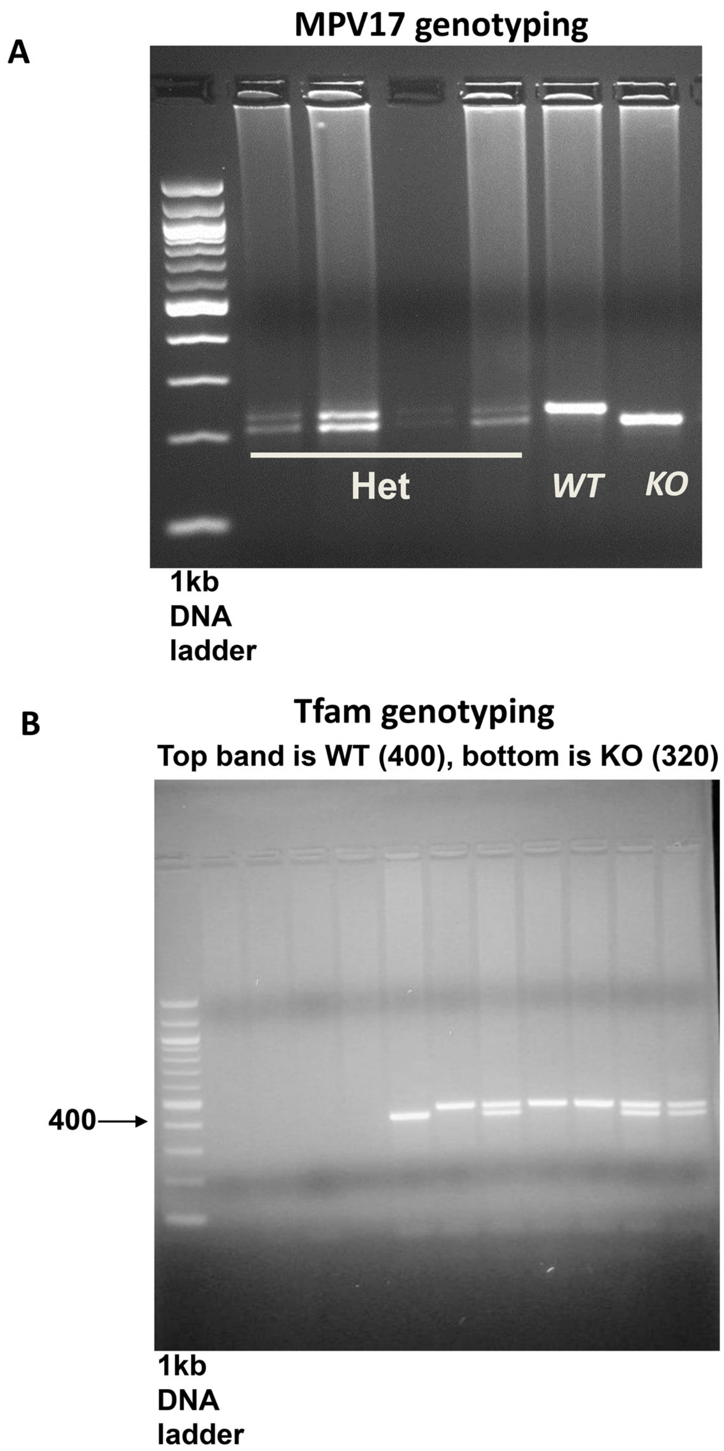

Supplementary Figure 1: Representative agarose (2%) gel image showing PCR analysis of primary esophageal epithelial cells derived from (**A**) WT and *MPV17*$^{+/-}$ or *MPV17*$^{-/-}$ mice and (**B**) from TFAM flox mice.

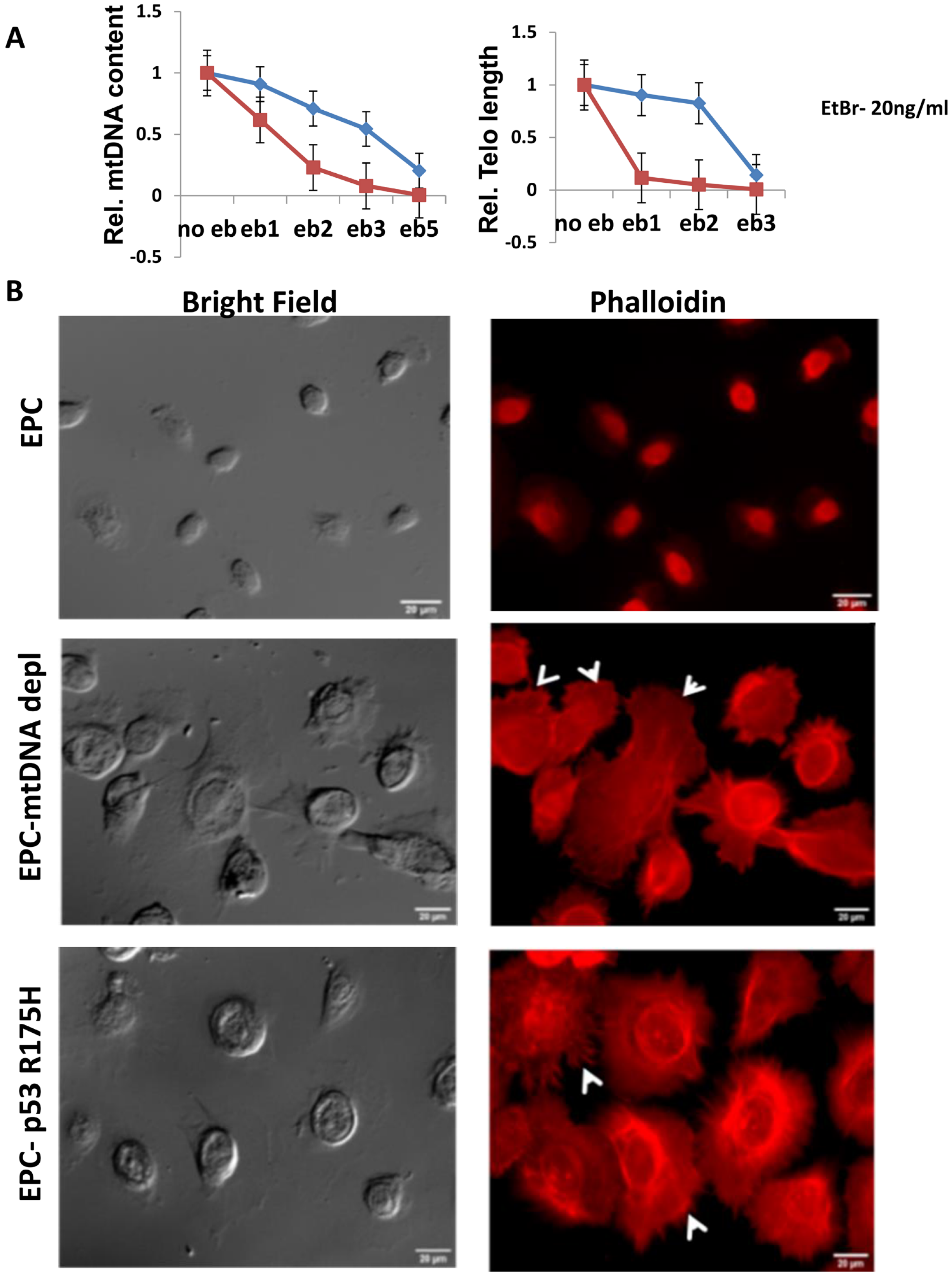

Supplementary Figure 2: (**A**) Left panel: Relative mtDNA content assessed from total DNA by real time PCR using primers for mtDNA coded gene (COXI) or nuclear coded single copy gene (CcOIVi1) in EPC2 cells (blue line) and EPC2 cells treated with 20 ng/ml EtBr (red line). Right panel: Relative telomere length analysis using genomic DNA isolated from EPC2 cells (blue line) and EPC2 cells treated with 20 ng/ml EtBr (red line). X-axis represents the number of cell generations of EtBr treatment. (**B**) Bright field (left panel) and phalloidin stained (right panel) images of EPC2 control, mtDNA depleted EPC2 and EPC2-TP53^{R175H} EECs. Arrows indicate the actin lamelipodia in mtDNA depleted EPC2 and EPC2-TP53^{R175H} EECs.

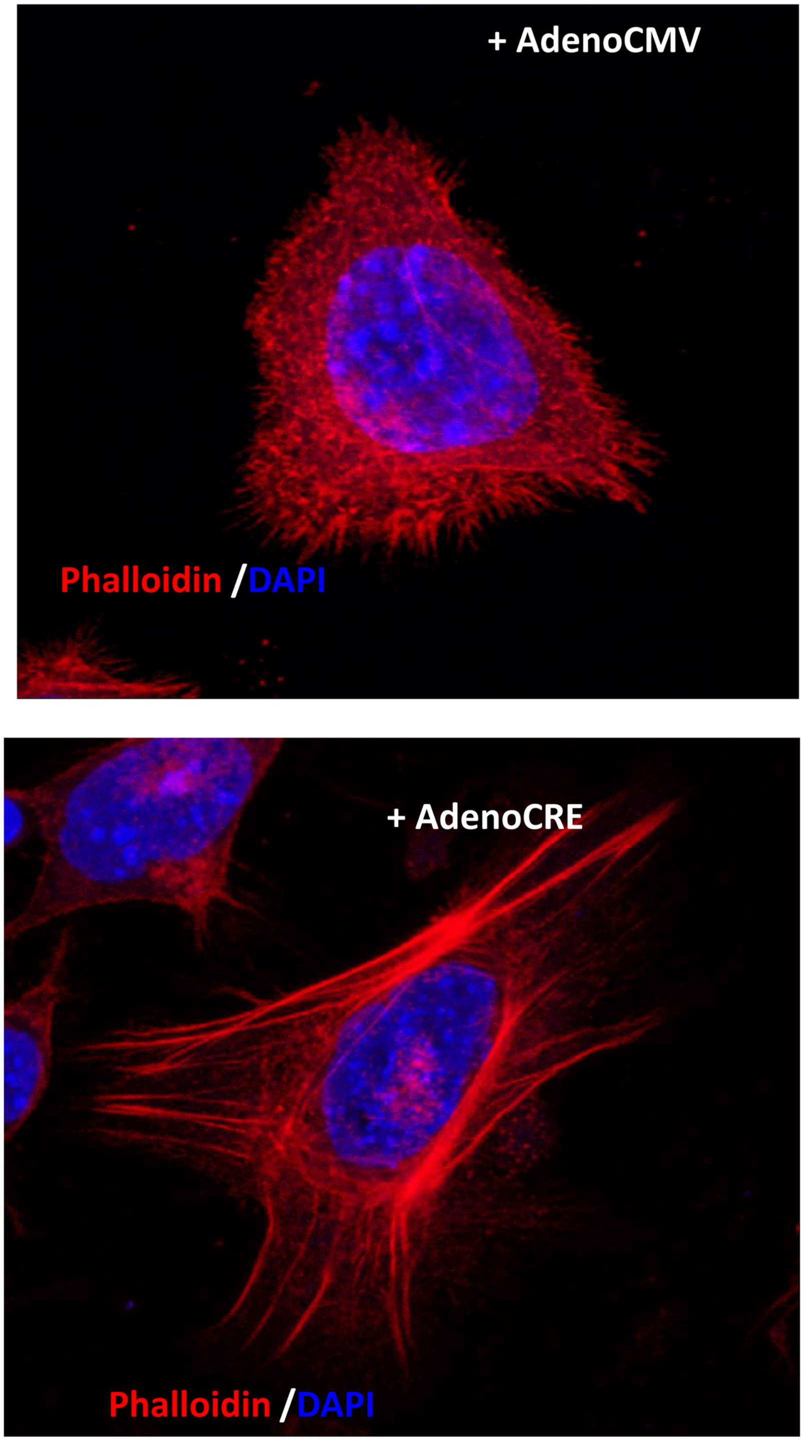

Supplementary Figure 3: Phalloidin staining of F-actin (red) and nucleus (DAPI, blue) in Tfam$^{+/fl}$ *ex vivo* transduced with either adeno CMV (control) or adeno CRE (Tfam$^{+/-}$) EECs imaged under 100x objective in Leica wide field microscope.

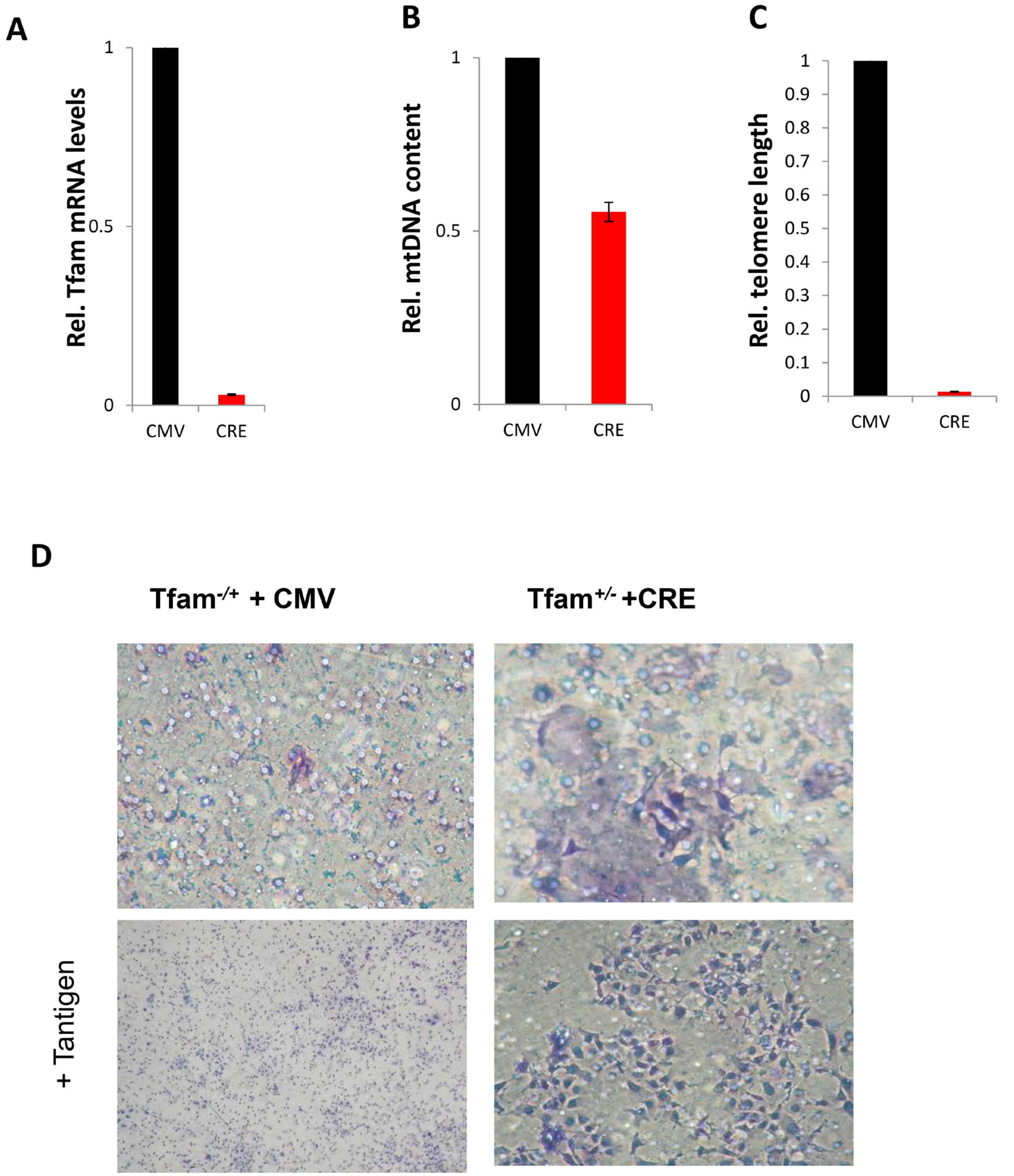

Supplementary Figure 4: (**A, B**) Real time PCR analysis of (**A**) Tfam mRNA transcript levels and (**B**) mtDNA content in EECs derived from Tfam$^{+/fl}$ *ex vivo* transduced with either adeno CMV (control) or adeno CRE. (**C**) Telomere length measured by real time PCR in EECs derived from Tfam$^{+/fl}$ *ex vivo* transduced with either adeno CMV (control) or adeno CRE (Tfam$^{+/-}$). (**D**) *In vitro* Matrigel invasion of EECs (1×10^4 cells) derived from Tfam$^{+/fl}$ *ex vivo* transduced with either adeno CMV (control) or adeno CRE (Tfam$^{+/-}$).

A

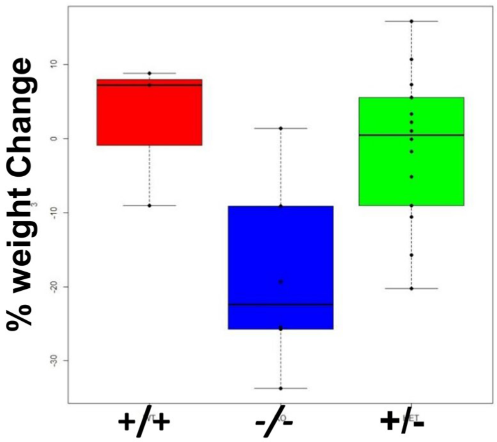

B

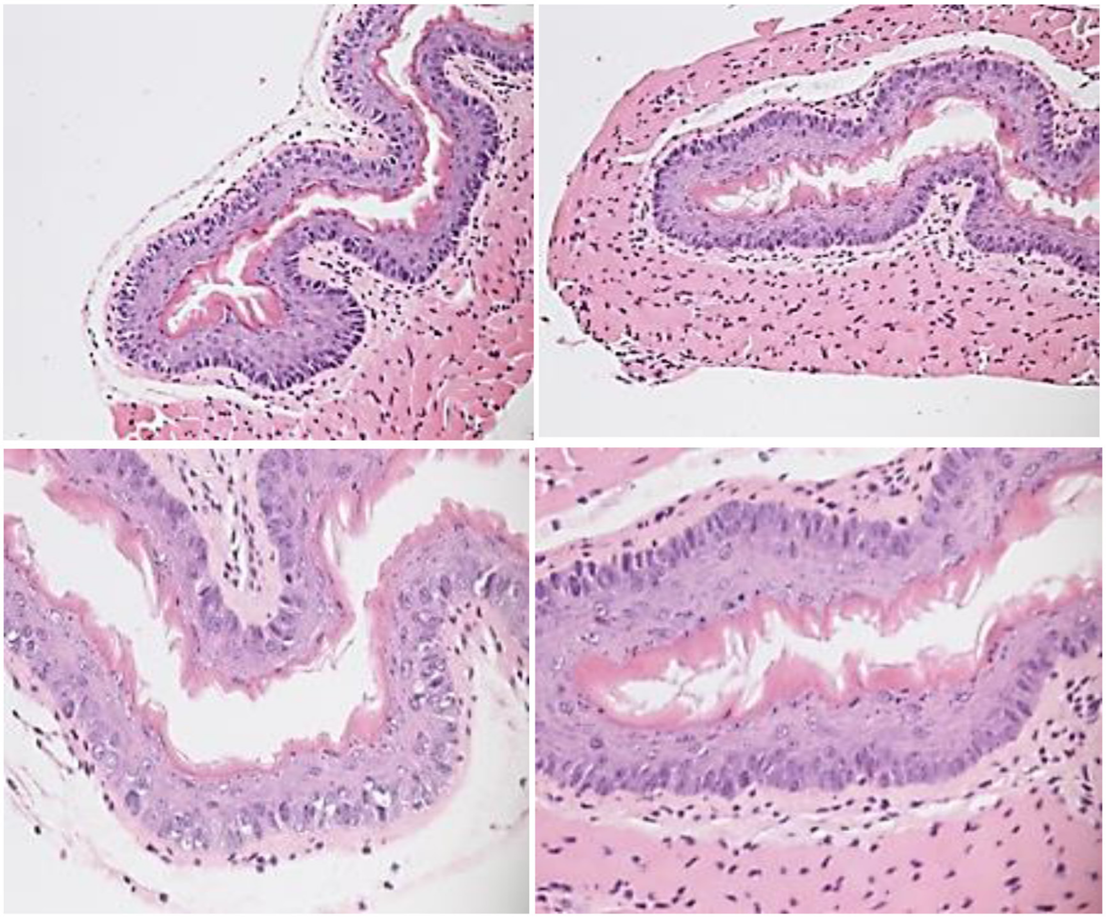

Supplementary Figure 5: 4NOQ treatment in *MPV*17 mice. (**A**) Body weight of WT (*n* = 10), *MPV17*$^{+/-}$ (*n* = 10) and *MPV17*$^{-/-}$ (*n* = 7) mice after 8 weeks of 4NQO treatment. (**B**) Hematoxylin and eosin stained sections of the esophageal lesions in 4NQO-treated *MPV17*$^{-/-}$ mice, Left: mild hyperplasia, Right: dysplasia.

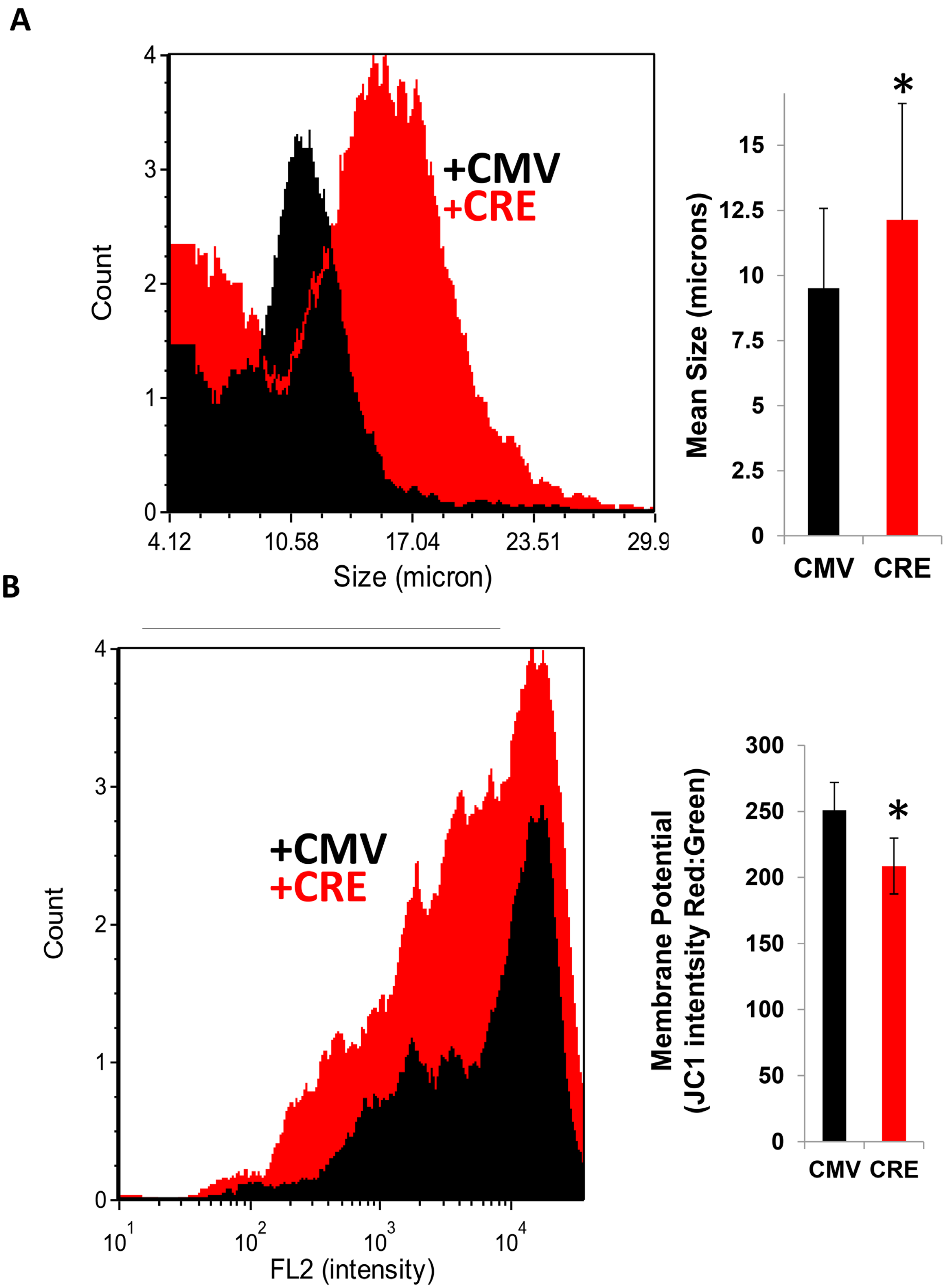

Supplementary Figure 6: (**A**) Cell size of TFAM$^{+/fl}$ +adeno CMV and TFAM$^{+/fl}$ +adeno CRE expressing EECs was measured under bright field using automated cell counter Cellometer Vision CBA (Nexcelom Biosciences, Lawrence, MA). (**B**) Mitochondrial membrane potential analysis analyzed on the Cellometer. Normal cells exhibit higher fluorescence intensities, whereas disrupted cells with decreased polarization in membrane potential exhibit reduced fluorescence. TFAM$^{+/fl}$ +adeno CRE expressing EECs show a significant reduction in red/green ratio indicating membrane potential disruption. Significance is indicated by $^{*}P < 0.05$.
